# Supplementary material for: Quantitative and Qualitative Identification of Bioactive Compounds in Edible Flowers of Black and Bristly Locust and Their Antioxidant Activity
Source: Biomolecules. 2020 Nov 26;10(12):1603. doi: 10.3390/biom10121603 (PMC7760478; doi:10.3390/biom10121603)
Supplement: Supplementary file 1 [file biomolecules-10-01603-s001.pdf]

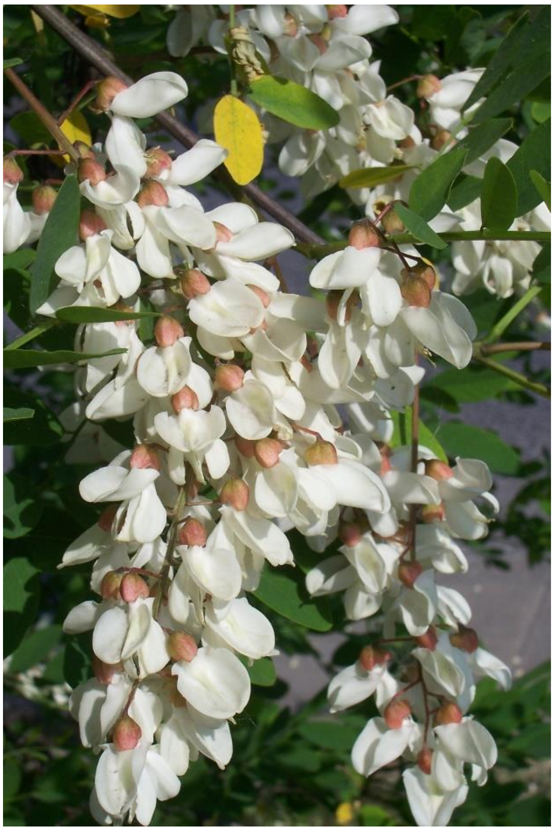

Photo 1. Flowers of *Robinia pseudoacacia* L. (Black locust)

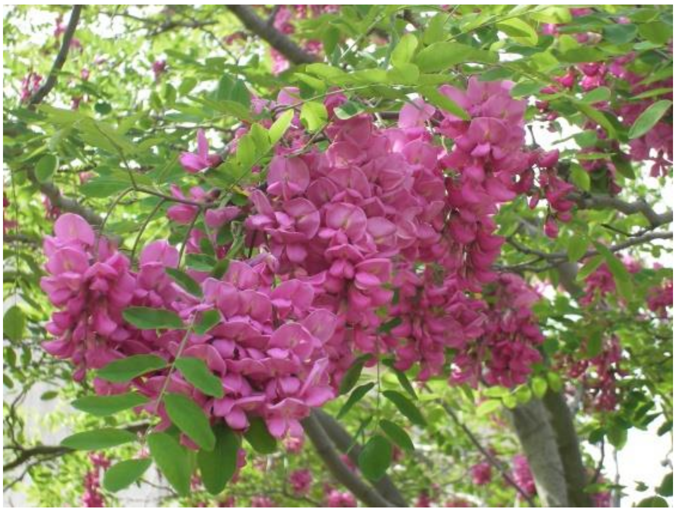

Photo 2. Flowers of *Robinia hispida* L. (Birstly locust)

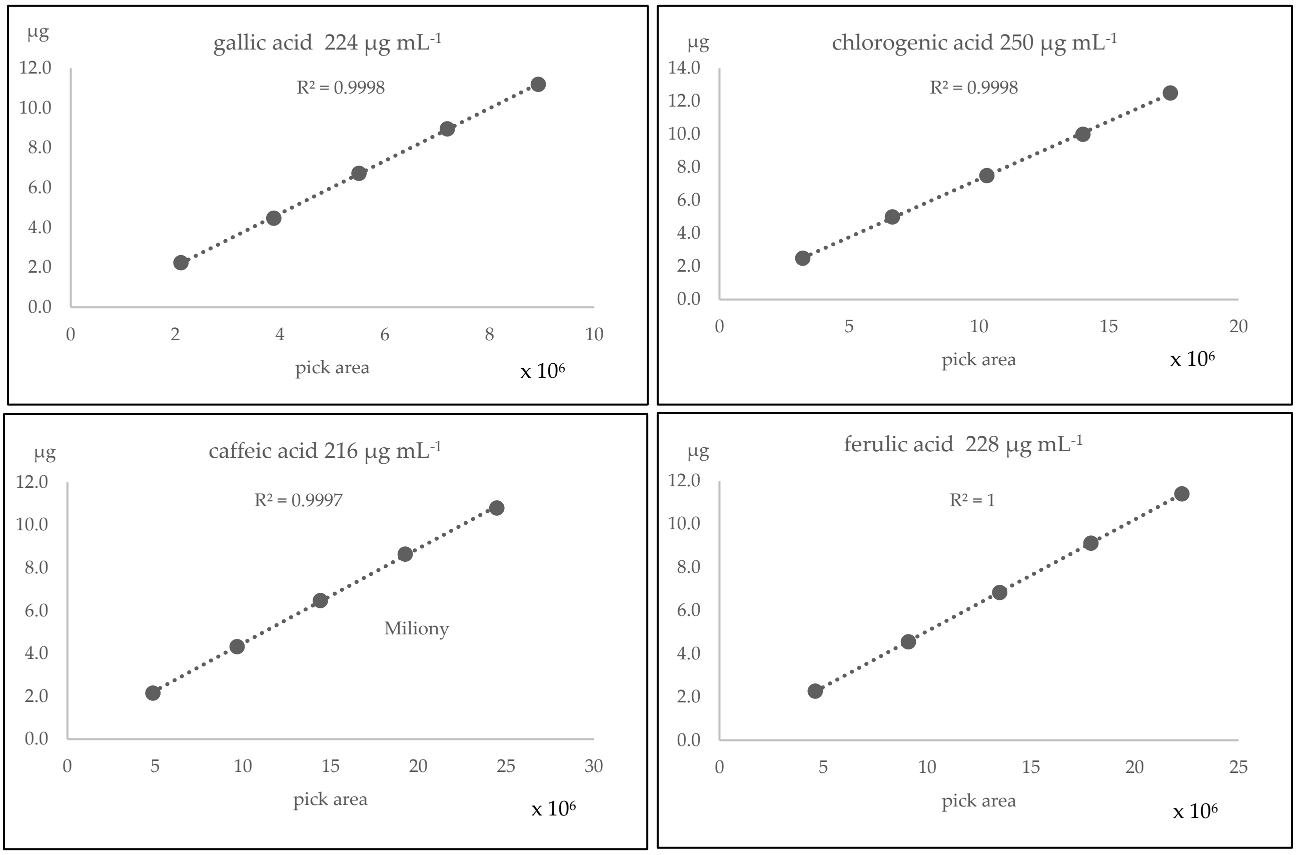

Figure S1. Standard curves for all phenolic acids identified in experiment with *Robinia* flowers

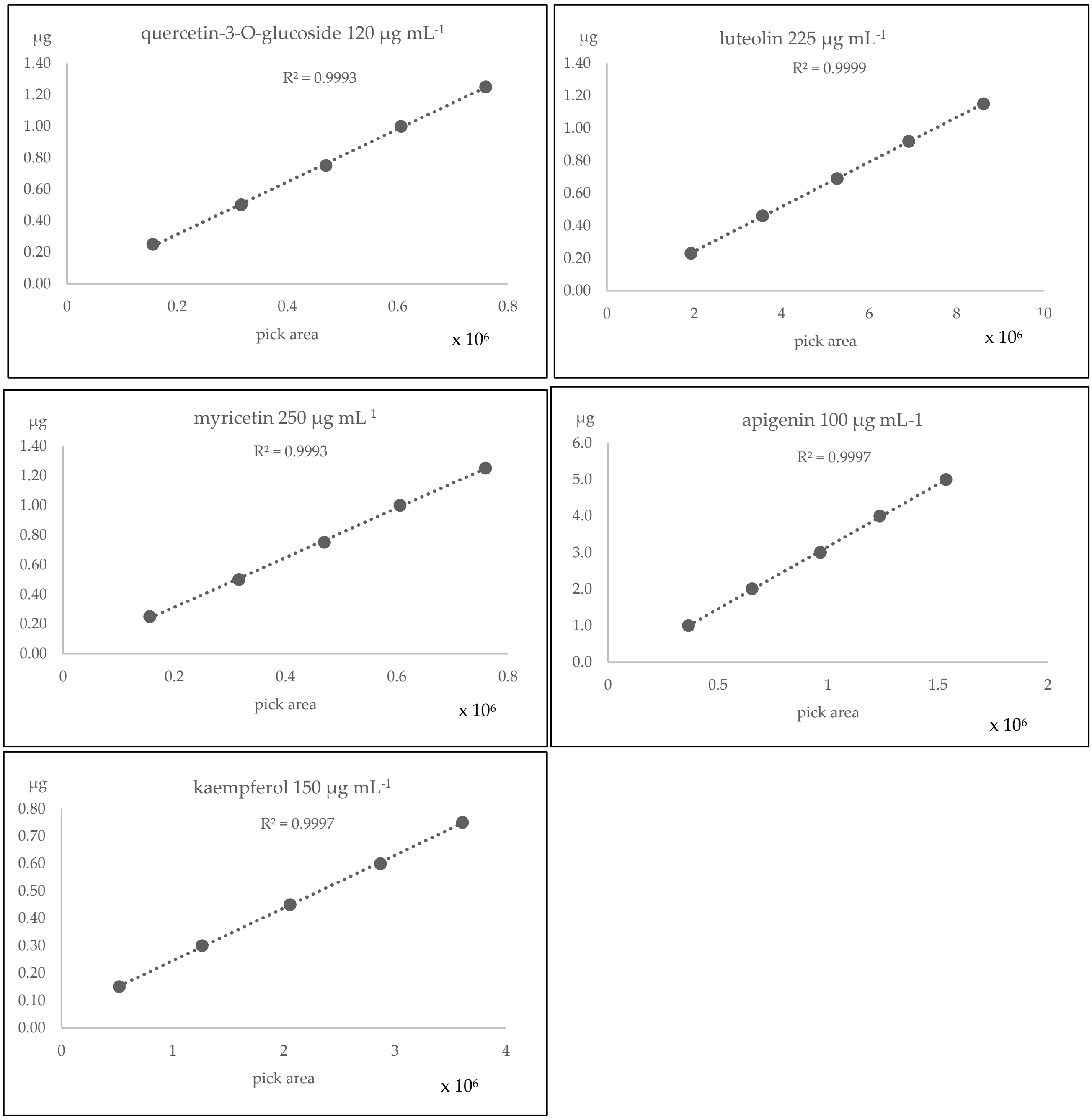

Figure S2. Standard curves for all flavonols identified in experiment with *Robinia* flowers

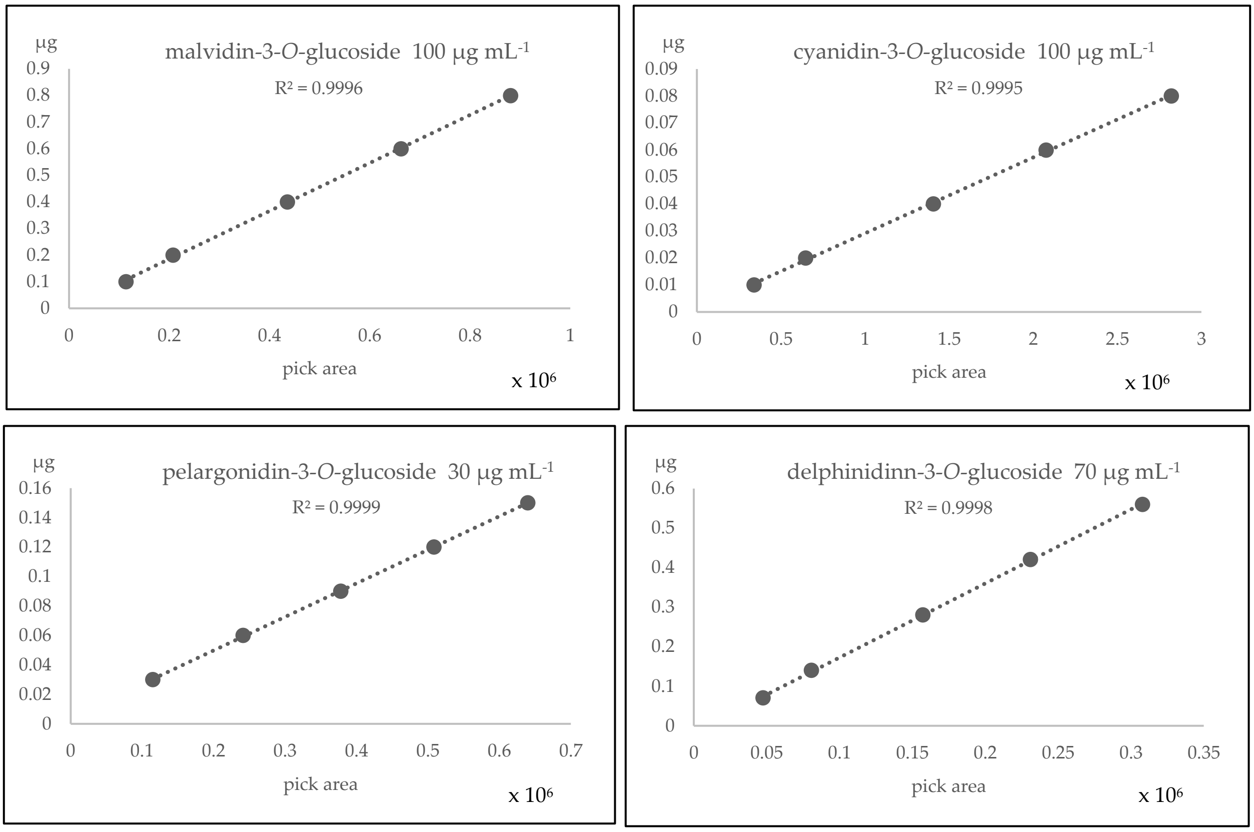

Figure S3. Standard curves for all anthocyanins identified in experiment with *Robinia* flowers

Figure S1. Standard curves for phenolic acids identified in experiment with *Robinia* flowers
